# Emx1 Is Required for Neocortical Area Patterning

**DOI:** 10.1371/journal.pone.0149900

**Published:** 2016-02-22

**Authors:** Adam M. Stocker, Dennis D. M. O’Leary

**Affiliations:** Molecular Neurobiology Laboratory, The Salk Institute, 10010 North Torrey Pines Road, La Jolla, California, 92037, United States of America; Instituto Gulbenkian de Ciência, PORTUGAL

## Abstract

Establishing appropriate area patterning in the neocortex is a critical developmental event, and transcription factors whose expression is graded across the developing neural axes have been implicated in this process. While previous reports suggested that the transcription factor Emx1 does not contribute to neocortical area patterning, those studies were performed at perinatal ages prior to the emergence of primary areas. We therefore examined two different Emx1 deletion mouse lines once primary areas possess mature features. Following the deletion of Emx1, the frontal and motor areas were expanded while the primary visual area was reduced, and overall the areas shifted posterio-medially. This patterning phenotype was consistent between the two Emx1 deletion strategies. The present study demonstrates that Emx1 is an area patterning transcription factor and is required for the specification of the primary visual area.

## Introduction

The neocortex is organized in the tangential domain into distinct functional fields, or areas, each of which area is responsible for processing modality specific information relayed from the periphery [1]. Among the key events in neural development is the specification of distinct functional areas within the neocortex; a process called neocortical area patterning [2–5].

Neocortical area patterning begins early in cortical development and is controlled by the interplay of intrinsic genetic regulation and extrinsic subcortical innervation. The extrinsic innervation of cortical areas by the dorsal thalamus helps to refine and establish mature, distinct functional areas [6,7]. Intrinsic regulation of neocortical patterning is characterized by transcription factors (TFs) expressed in neural progenitors in gradients along the medial-lateral and anterior-posterior axis [2,5]. Most of these TFs are in turn modulated and established by morphogens [3,4]. The TFs are expressed in neural progenitors early in neurogenesis, and most have maintained expression throughout neurogenesis [2,4].

Thus far, only four area patterning TFs have been identified: Emx2 [8,9], COUP-TF1 (NR2F1) [10], Sp8 [11,12], and Pax6 [8]. The highest level of Emx2 expression is located posterio-medially and it specifies posterior areas, in particular visual areas [13,14]. COUP-TF1 is expressed highest posterior-laterally and promotes posterior fates by repressing anterior identities [15]. Sp8 is expressed highest antero-laterally and specifies anterior areas [11,12]. Pax6 is expressed highest antero-laterally and specifies anterior areas, in particular somatosensory areas [16,17]. The interplay of these TFs impart areal fates to neural progenitors and their progeny [2,5].

Emx1 is a homeodomain transcription factor expressed in a gradient, similar to that of Emx2, across cortical neural progenitors where the highest level of expression is located posterio-medially and the lowest antero-laterally [18–21]. Early experimental approaches examined gross changes of gene expression patterns in tissue of perinatal homozygous Emx1 deletions, and all of these studies found no evidence that Emx1 participates in area patterning [16,22–24]. However, subsequent examination of Emx1 mutant cortices at postnatal stages is lacking. This is an important omission, since hallmark features of areas emerge at postnatal time points after thalamic projections have reached the cortex and been refined into sharp borders [2,6]. Previous approaches were therefore limited by the early analysis and did not examine the effects of Emx1 deletion on areas that possess many mature features.

Because Emx1 is a homeodomain transcription factor expressed in a gradient across neural progenitors, and the previous studies possessed obvious limitations, we hypothesized that Emx1 may be required for neocortical area patterning. If so, then the deletion of Emx1 from the cortex would cause area patterning changes similar to Emx2 heterozygotes, where anterior areas are expanded, and posterior areas, in particular the primary visual area, are reduced.

## Materials and Methods

### Mice

All experiments were approved and conducted following the guidelines of the Institutional Animal Care and Use Committee at the Salk Institute (protocol number: 12–00016) and were in full compliance with the guidelines of the US National Institutes of Health for the care and use of laboratory animals. Analyses were done independent of sex and included both male and female mice according to Mendelian ratios.

Emx1 knock out mice (Mouse Genome Informatics ID: 1857433; Emx1^-/-^) were previously generated [25], and were a generous gift from the Rubenstein laboratory. The Emx1 knock in mice (Mouse Genome Informatics ID: 1928281; Emx1^cre/+^), where Cre-recombinase has been knocked into the Emx1 locus replacing and effectively deleting Emx1 [26], were obtained from the RIKEN Institute (Japan). Emx1 knock in mice were crossed to the conventional knock out line to generate the new deletion strategy (Emx1^cre/-^), providing an alternative approach to the conventional line and maintaining heterozygous Cre-recombinase expression consistent with previous studies [26].

### Immunohistochemistry

Postnatal day 7 (P7) mice were perfused by cardiac infusion of 4% buffered paraformaldehyde (PFA) following IP administration of the anesthetic Avertin (2, 2, 2-Tribromoethanol) at a dose in excess of 0.017ml/g body weight, and then the brains were removed. For tangential sections cortical hemispheres were dissected, post-fixed between glass slides, then cryoprotected in sucrose/phosphate buffered saline (PBS). Tangential sections were cut at 50μm from flattened hemispheres and used for in situ hybridization or immunohistochemistry, as described previously [6,13]. For sagittal sections a mid-sagittal cut was made to separate the two hemispheres, which were post-fixed overnight, cryoprotected in sucrose/PBS and frozen in Tissue-Tek OCT compound (Sakura). Sagittal sections were cut at 20μm from hemispheres with a cryostat (Leica) and used for in situ hybridization or immunohistochemistry. For immunostaining endogenous peroxidase activity was quenched by incubation with 0.06% H_2_O_2_/PBS for 30 minutes, sections were washed with PBS, incubated in blocking solution (3% bovine serum albumin in PBS), then incubated in blocking solution with a primary antibody against serotonin (1:50,000; Immunostar) overnight at 4°C. Sections were rinsed in PBS, incubated for 2 hours in biotin-conjugated anti-rabbit IgG secondary antibody (1:200; Jackson ImmunoResearch), and rinsed again. Sections were developed following the standard DAB (di-amino-benzidine) colorimetric reaction (Elite ABC Kit; Vector).

### In situ hybridization

Sagittal and tangential sections were collected as described above, and in situ hybridization was carried out as previously described [6,13,17] with slight modifications. Antisense RNA probes were labeled using a DIG (digoxigenin) RNA labeling kit (Roche) and previously described cDNA templates. Probes for RORβ (Entrez gene ID: 225998), Cad8 (Entrez gene ID: 12564), Lmo4 (Entrez gene ID: 16911), and Igfbp5 (Entrez gene ID: 16011) were described previously [6]. Sections were dried, post-fixed, rinsed in PBS, digested with 3μg/mL Proteinase K for 3 minutes, fixed again with 4% PFA containing 0.2% glutaraldehyde, then rinsed with PBS. Prehybridization was done for 1 hour at 65°C in hybridization buffer: 50% formamide, 5x saline-sodium citrate buffer (SSC, pH 4.5), 5mM Ethylenediaminetetraacetic acid (EDTA), 1mg/mL 3-[(3-cholamidopropyl) dimethylammonio]-1-propanesulfonate (CHAPS), 10mg/mL Boehringer’s blocking powder, 200μg/mL Heparin, 0.1% Tween-20, 500μg/mL yeast transfer RNA (tRNA). Sections were incubated overnight in hybridization buffer with 1–3μg/mL of DIG-labeled probe at 65°C. Sections were then washed at 65°C with solution A containing 50% formamide and 2x SSC (pH 4.5), then at room temperature with solution B containing 50mM Tris-HCl (pH 7.5), 0.15M NaCl, 10mM KCl, and 0.5% Tween-20. Sections were blocked in solution B containing 15% fetal calf serum (i.e. blocking solution) for 2 hours, then incubated overnight at 4°C in blocking solution containing an alkaline-phosphatase-conjugated anti-DIG antibody (1:2,000; Roche). Sections were washed in solution B, then in solution C containing 0.1M Tris-HCl (pH 9.5), 0.1M NaCl, 50mM MgCl_2_, and 0.2% Tween-20. After washing, the color reaction was performed using 0.35mg/mL Nitrotetrazolium Blue Chloride (NBT) and 0.175mg/mL 5-Bromo-4-Chloro-3-Indoxyl phosphate, p-toluidine salt (BCIP) in solution C at room temperature for 1 to 12 hours, and stopped with PBS containing 0.1% Tween-20 (PBT).

Whole mount in situ hybridization was also performed as described previously [6,13], with slight modifications. Fresh brains were dissected and then meninges were removed from dissected brains prior to immersion-fixation in 4% PFA at 4°C for 2 days. Brains were washed in PBT, saturated step-wise in methanol, rehydrated in a reverse step-wise fashion, and then washed again in PBT. Brains were bleached with 6% H_2_O_2_ in PBT to quench endogenous peroxidase activity then washed again in PBT. Brains were digested with 3μg/mL Proteinase K for 30 minutes, fixed again with 4% PFA containing 0.2% glutaraldehyde, then rinsed with PBT. Prehybridization was done for 1 hour at 70°C in whole mount hybridization buffer: 50% formamide, 5x SSC (pH 4.5), 1% sodium dodecyl sulfate (SDS), 50μg/mL Heparin, 50μg/mL yeast tRNA. Brains were incubated overnight at 70°C in whole mount hybridization buffer with 0.5μg/mL of DIG-labeled probe. Following hybridization, brains were washed at 70°C with solution D containing 50% formamide, 5x SSC (pH 4.5), and 1% SDS, then at 65°C with solution A (detailed above). Brains were washed at room temperature with a solution E containing 25mM Tris-HCl (pH 7.5), 135mM NaCl, 275μM KCl, and 0.1% Tween-20. Brains were blocked in solution E containing 10% fetal calf serum and 10% bovine serum albumin (i.e. blocking solution) for 2 hours, then incubated overnight at 4°C in blocking solution containing an alkaline-phosphatase-conjugated anti-DIG antibody (1:2,000; Roche). The next day brains were washed in solution E, then in solution C (detailed above). After washing, the color reaction was performed using 0.35mg/mL NBT and 0.175mg/mL BCIP in solution C at room temperature for 1 to 12 hours, and stopped with PBT.

### Axonal tracing

Post natal day 21 (P21) mice were perfused with 4% PFA, then brains were removed, cut mid-sagittally to separate the two hemispheres, and post-fixed for 2 days. Axonal labeling was performed as described previously [15,27], with slight modifications. To anterogradely label the thalamocortical afferent (TCA) projection, a coronal cut was made at the caudal edge of the diencephalon, to reveal the dorsal thalamus. A crystal of 1,1'-Dioctadecyl-3,3,3',3'-Tetramethylindocarbocyanine Perchlorate (DiI, Molecular Probes) was placed into the dorsal lateral geniculate nucleus (dLG) to label visual TCA projections into the visual cortex, then incubated at 37°C for 4 months in 4% PFA. After incubation, brains were embedded in 5% low-melting agarose (RPI Corp), cut into 150μm thick sagittal sections on a vibratome (Leica), counterstained with DAPI (4’-6-diamidino- 2-phenylindole) (Vector Labs), mounted in PBS/Glycerol, and photographed under fluorescent light. Each tracing experiment was repeated at least seven times and the results were reproducible.

### Image acquisition, image analysis, and statistical analysis

Immunofluorescent and brightfield images were captured using an Olympus BX61 microscope, QImaging Retiga-2000DC camera, and the Metamorph Advanced (Molecular Devices) program. Images of whole mount in situs and tangential sections were captured using a Zeiss Stemi SV11 microscope, AxioCam MRc5 camera (Zeiss), and the AxioVision (Zeiss) program. Image analysis and measurements for all genotypes were performed using ImageJ (NIH) [28] (datasets are included in S1 Appendix), and statistical analysis for all collected data was performed using R (CRAN, http://cran.r-project.org) [29].

## Results

The hypothesis that Emx1 (Entrez gene ID: 13796) is required for neocortical area patterning predicts that the size of the anterior and posterior areas will change, most notably that the primary visual area (V1) will be reduced in size following Emx1 deletion. The expression of Emx1 is restricted to the neocortex [20,21], thereby eliminating the concern for off-target effects in other tissues often associated with constitutive knock out approaches [30–33]. Accordingly, the constitutive knock outs previously utilized to examine Emx1 function [25] can be employed here as the knock out animals survive through adulthood. Two lines generated previously were used in this study: a constitutive deletion (Emx1^-/-^) [25]; and an effective Emx1 deletion where Cre-recombinase has been knocked into the Emx1 locus [26]. The Cre-recombinase knock in line was crossed to the constitutive deletion line creating an additional deletion approach (Emx1^cre/-^) possessing heterozygous Cre-recombinase expression, which is consistent with previous use of this cre line [26]. Both Emx1 deleted genetic lines utilized in this study, as reported previously, were viable and survive through adulthood [25,26].

### Emx1 deletion reduces V1 size but does not alter lamination

Emx2 has been demonstrated previously to specify the primary visual area (V1) [13,14]. Emx1 is closely related to Emx2 and expressed in the same gradient across neural progenitors in the ventricular zone from early in cortical development [19–21]. Therefore, we examined the effect that Emx1 deletion has on the specification of V1. Established areal genetic markers with expression that delineates V1 allowed us to examine qualitative changes in postnatal V1 size following the deletion of Emx1. These marker analyses were performed on sagittal sections of post natal day 7 (P7) brains, when primary sensory area size appears mature [2,6], to elucidate V1 size and cortical lamination.

**Fig 1:**
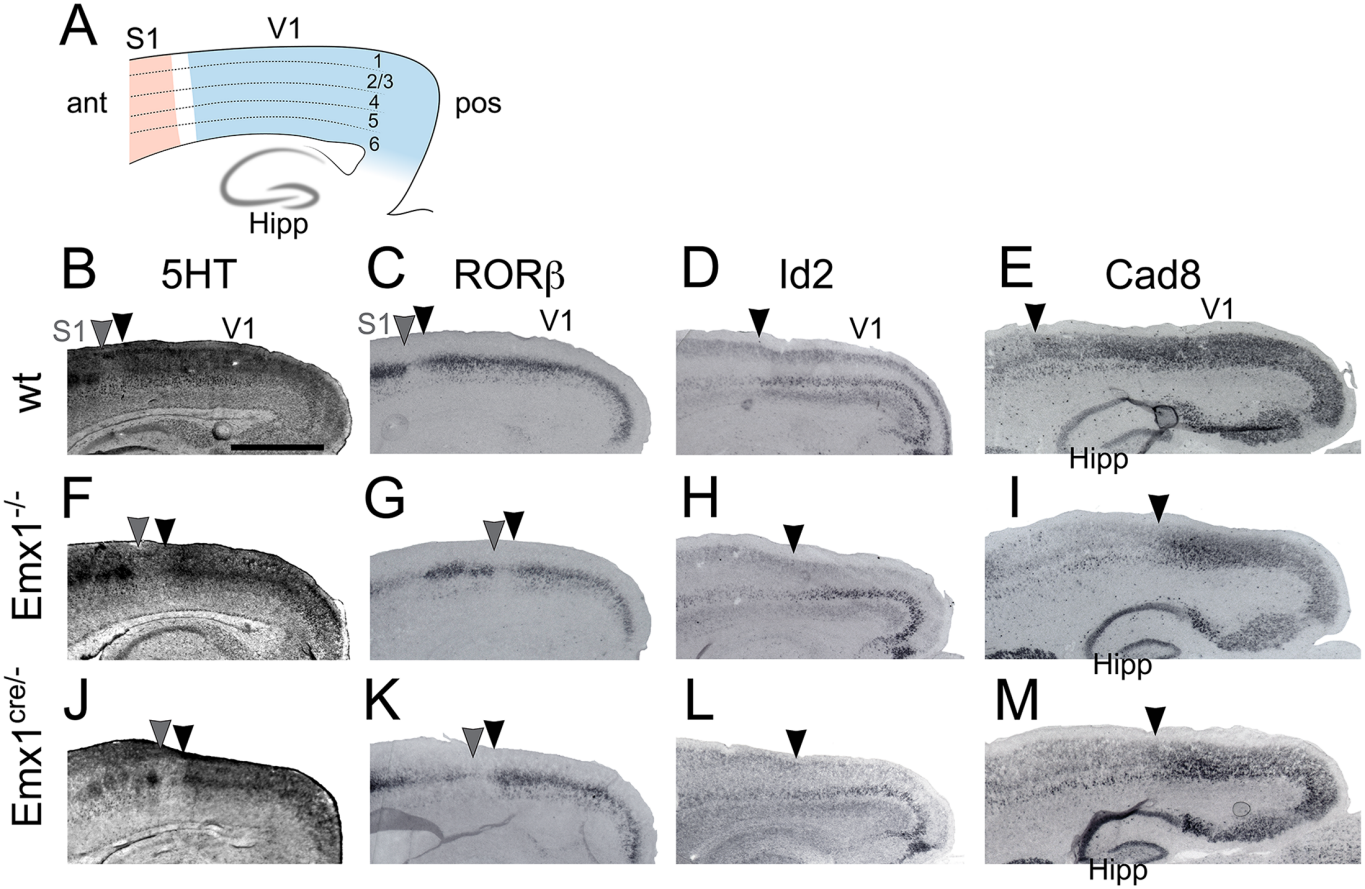
Gene expression demonstrates a reduction in V1 size with normal laminar expression following Emx1 deletion. Immunostaining and in situ hybridization for several markers was performed on sagittal sections of P7 cortices. (A) Schematic of a sagittal section detailing the cortical layers and location of the hippocampus (Hipp). Stained sections of wt (B–E), Emx1^-/-^ (F–I), and Emx1^cre/-^ (J–M) have been cropped to highlight changes in V1. (B, F, J) 5HT immunostaining revealed layer 4 of V1 and S1. Compared to wt, Emx1 deletions (Emx1^-/-^ and Emx1^cre/-^) possessed a smaller, posteriorly shifted V1 (black arrowheads denote anterior edge of V1, gray arrowheads denote posterior edge of S1). (C, G, K) RORβ is expressed in layer 4 of primary sensory areas. In Emx1 deletions the posterior shift of the anterior edge of V1 (black arrowheads) and posterior edge of S1 (gray arrowheads) can be appreciated, showing the reduction in V1 size. (D, H, L) Id2 is expressed in layer 5 of V1 and also showed a posterior shift of the anterior edge of V1 in Emx1 deletions (arrowheads). (E, I, M) Cad8 is expressed in layers 2/3 of V1, and in Emx1 deletions the anterior edge of V1 (arrowheads) was shifted posteriorly, and V1 size was reduced. Cad8 is also expressed in the hippocampus, which is ventral to the cortex and provides a stable landmark for comparison between genotypes. All of the stains presented demonstrated the reduction of V1 in the sagittal plane, as well as the posterior shift of V1. However all of these markers were expressed in the appropriate cortical layers. Anterior is to the left, dorsal to the top. V1, primary visual area; S1, primary somatosensory area; Hipp, hippocampus; ant, anterior; pos, posterior. Scale bar, 1.0 mm. Bar in B applies to all panels.

Serotonin (5HT) immunostaining was used to visualize thalamocortical terminations in layer 4 of V1 [34]. In both Emx1 deletion approaches, apparent V1 size was reduced and the anterior edge of V1 was noticeably shifted posteriorly compared to controls (Fig 1B, 1F and 1H). The RAR-related orphan nuclear receptor (RORβ) is also expressed in layer 4 of V1 [35,36], and it too demonstrated that V1 was reduced with the anterior edge shifted in Emx1 deletions (Fig 1C, 1G and 1K). The dominant negative basic helix-loop-helix protein (Id2) is expressed in layer 5 of V1 [37]. Id2 staining also indicated that V1 was smaller in both Emx1 deletion approaches, and that the anterior edge has been shifted posteriorly (Fig 1D, 1H and 1L). The calcium-dependent cell adhesion protein cadherin8 (Cad8) is expressed in layers 2/3 in V1 [6,19]. Cad8 is also expressed in the hippocampus, specifically in the pyramidal cell layers of CA1 –CA3 and in the granule cell layer of the dentate gyrus [38]. This hippocampal expression provided an anatomical landmark to compare between genotypes. Cad8 staining showed there was a slight posterior shift of the hippocampus in Emx1 deletions, however relative to the hippocampus the anterior edge was also shifted posteriorly and V1 was reduced in size in Emx1 deletions similar to the other stains on sagittal sections (Fig 1E, 1I and 1M).

While the area denoted by expression of these genes was reduced, which indicated that V1 was reduced in size, all were expressed in their appropriate cortical layers. These results demonstrated that the size of V1, as measured by gene expression, has been reduced and the anterior edge shifted posteriorly. This reduction and shift was still present when compared to the location of another anatomical structure. However no other changes were apparent from these qualitative analyses, in particular lamination was normal, suggesting that the changes were the result of a change in neocortical area patterning.

### Emx1 deletion changes gene expression patterning

**Fig 2:**
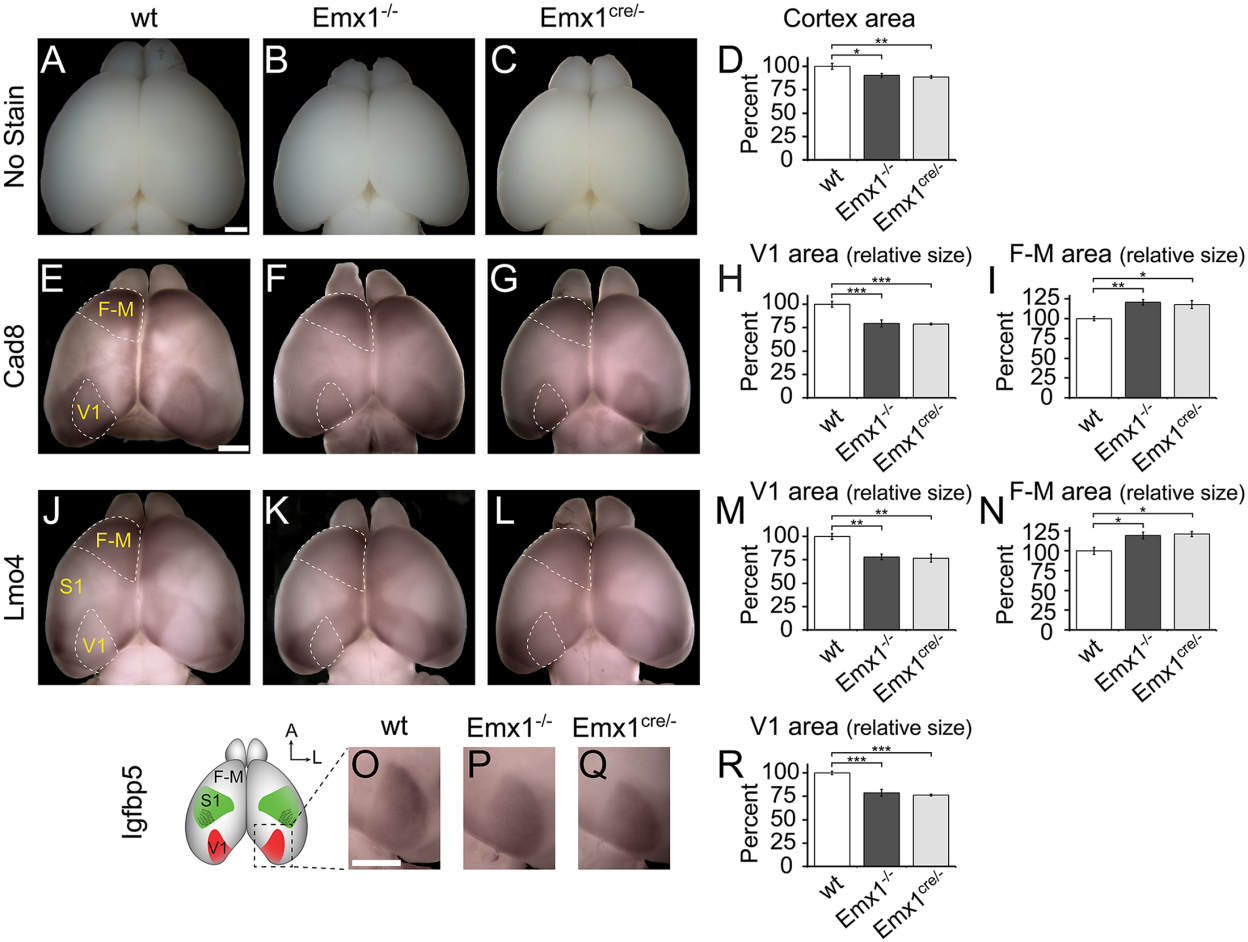
Gene expression demonstrates reduction in V1 and expansion of F-M in Emx1 deleted animals. Whole mount in situ hybridization (WMISH) was performed on P7 cortices to observe changes in area patterning following Emx1 deletion. (A–C) Examples of fixed, unstained P7 cortices from each genotype. (D) Analysis of cortical area demonstrated that both Emx1 deletions possessed cortices smaller than wt. (E–G) Cad8 expression labels F-M and V1 (both outlined). (H) V1 area was normalized by total cortical area, and the relative V1 area size in both Emx1^-/-^ and Emx1^cre/-^ cortices, as measured by Cad8, was significantly smaller than wt. (I) F-M was normalized by total cortical area, and the relative F-M area size in both Emx1^-/-^ and Emx1^cre/-^ cortices, as measured by Cad8, was significantly larger than wt. (J–L) Lmo4 marks V1 (outlined) and S1 with lower expression relative to surrounding regions and was highly expressed in F-M areas (outlined). (M) Lmo4 V1 relative area in both Emx1^-/-^ and Emx1^cre/-^ cortices was significantly smaller than wt. (N) Lmo4 F-M relative area in both Emx1^-/-^ and Emx1^cre/-^ cortices was significantly larger than wt. (O–Q) Igfbp5 expression labels V1, thus only the posterio-medial portion of P7 cortices are shown, as indicated in the schematic. (R) Igfbp5 V1 relative area in both Emx1^-/-^ and Emx1^cre/-^ cortices was significantly smaller than wt. Data presented as percentage of wt. V1, primary visual area; S1, primary somatosensory area; F-M, frontal and motor areas. *P<0.05; **P<0.01; ***P<0.001; Tukey’s HSD post hoc test. Scale bar, 1.0 mm. Bar in A applies to A–C, E–G, J–L. Bar in O applies to O–Q.

The changes in V1 area markers were consistent with changes observed in Emx2 heterozygous mutants [13]. To examine area patterning in the entire neocortex, area markers were employed with whole mount in situ hybridization (WMISH) to observe potential areal changes in the entire intact neocortex. Total cortical size was quantified for each genotype (wt, Emx1^-/-^, Emx1^cre/-^), and we found that the differences in cortical size between the three genotypes were statistically significant (one way ANOVA, F_(2,46)_ = 6.87; P = 0.0025) (Fig 2A–2D). Specifically, Emx1^-/-^ cortices were 9.57% ±2.13% (±SEM) smaller than controls (Tukey’s HSD post-hoc analysis, P = 0.0192) and Emx1^cre/-^ cortices were 11.42% ±1.50% smaller than controls (P = 0.0032), however there were no significant differences between the Emx1 deletions (P = 0.8488) (Fig 2D).

Cad8 expression in WMISH delineates V1 as well as frontal-motor areas (F-M) [6,19] (Fig 2E–2G). Qualitatively, Cad8 expression in WMISH demonstrated similar reductions in V1 size compared to the sagittal stains (Fig 1); however, the sensory areas appeared normal in shape (Fig 2E–2G). For quantification of these changes, individual area size was normalized by the total cortical size yielding the relative size that corrected for any differences caused by the reduction in total cortical size observed in mutants. The relative size of V1 as measured by Cad8 expression was significantly different between genotypes (F_(2,13)_ = 19.21; P = 0.0001; wt n = 7, Emx1^-/-^ n = 4, Emx1^cre/-^ n = 5) (Fig 2H). Cad8 expression demonstrated that V1 relative to total cortical area was reduced 20.51 ±3.68% in Emx1^-/-^ (P = 0.0007) and 21.15 ±0.98% in Emx1^cre/-^ (P = 0.0003) compared to controls, while there were no differences between the Emx1 deletions (P = 0.9890) (Fig 2H).

The Cad8 results show that both Emx1 mutants had a statistically significant reduction in relative V1 size. Anterior areas, specifically frontal-motor areas (F-M), were also examined to see if any changes were present following Emx1 deletion. Cad8 expression is also present throughout F-M [6,19], and showed that F-M was qualitatively larger in Emx1 deletions (Fig 2E–2G). Again for the quantification of these changes area size was normalized by the total cortical size. The relative size of the F-M area as measured by Cad8 expression was significantly different between genotypes (F_(2,25)_ = 6.797; P = 0.0044) (Fig 2I). Cad8 expression demonstrated that F-M relative to total cortical size was increased 20.73 ±3.58% in Emx1^-/-^ (P = 0.0085) and 17.79 ±5.05% in Emx1^cre/-^ (P = 0.0294) compared to controls, while there were no differences comparing the two different Emx1 deletions (P = 0.9926) (Fig 2I).

The expression of transcription factor LIM domain only 4 (Lmo4) viewed by WMISH highlights area boundaries with reduced expression relative to surrounding regions in V1 and S1, and increased expression in F-M [39]. Qualitatively, Lmo4 expression in WMISH (Fig 2J–2L) demonstrated a reduction in V1 size after Emx1 deletion, as well as a posterior shift of the primary somatosensory area (S1). The relative V1 size measured by Lmo4 expression was significantly different between genotypes (F_(2,15)_ = 12.51; P = 0.0006; wt n = 5, Emx1^-/-^ n = 8, Emx1^cre/-^ n = 5) (Fig 2M). Lmo4 expression demonstrated that the relative size of V1 was reduced 21.99 ±3.02% in Emx1^-/-^ (P = 0.0012) and 23.39 ±4.34% in Emx1^cre/-^ (P = 0.0018) compared to controls, while there were no differences comparing the two Emx1 deletions (P = 0.9565) (Fig 2M).

Lmo4 expression correspondingly demonstrated an expansion of F-M size in Emx1 deletions (Fig 2J–2L). The relative F-M size as measured by Lmo4 expression was significantly different amongst genotypes (F_(2,15)_ = 6.957; P = 0.0073) (Fig 2N). Lmo4 expression demonstrated that F-M relative to total cortical size was increased 19.36 ±4.10% in Emx1^-/-^ (P = 0.0125) and 21.25 ±3.32% in Emx1^cre/-^ (P = 0.0135) compared to controls, while there were no differences between the Emx1 deletions (P = 0.9445) (Fig 2N).

Expression of insulin-like growth factor binding protein-5 (Igfbp5) is restricted to V1 in superficial cortical layers [6,40]. WMISH against Igfbp5 also demonstrated that V1 was reduced in Emx1 deleted cortices (Fig 2O–2Q). The relative V1 size measured by Igfbp5 expression was significantly different between genotypes (F_(2,11)_ = 26.71; P < 0.0001; wt n = 5, Emx1^-/-^ n = 5, Emx1^cre/-^ n = 4) (Fig 2R). Igfbp5 expression demonstrated that the V1 size relative to total cortical size was reduced 21.27 ±3.60% in Emx1^-/-^ (P = 0.0001) and 23.69 ±0.94% in Emx1^cre/-^ (P = 0.0002) compared to controls, while there were no differences comparing the two Emx1 deletions (P = 0.7931) (Fig 2R).

The expression of different classes of established area markers demonstrated that V1 was reduced, when Emx1 was deleted from the developing neocortex. These markers also demonstrated that F-M is expanded in Emx1 deletions. Furthermore, the quantitative results were consistent between the two genetic deletion lines and across the utilized markers.

### Emx1 deletion changes the patterning of thalamic terminations

There are several features that distinguish individual functional areas, including distinct gene expression profiles [3–5]. Another key distinguishing feature is that of unique input and output projections [2–4]. Primary sensory areas receive modality-specific input that is relayed from the periphery through the dorsal thalamus [2,41]. Given the changes observed via gene expression, we examined thalamic projections in Emx1 deletions for any potential changes.

Serotonin (5HT) is expressed in thalamocortical terminations in layer 4 of primary sensory areas [34]. Immunostaining against 5HT delineated primary sensory areas that could be best visualized on tangential sections of flattened cortex. As detailed above, RORβ was also expressed in layer 4 of the primary sensory areas [35,36], which provided a means to qualitatively compare gene expression and thalamic input in the same type of preparation.

**Fig 3:**
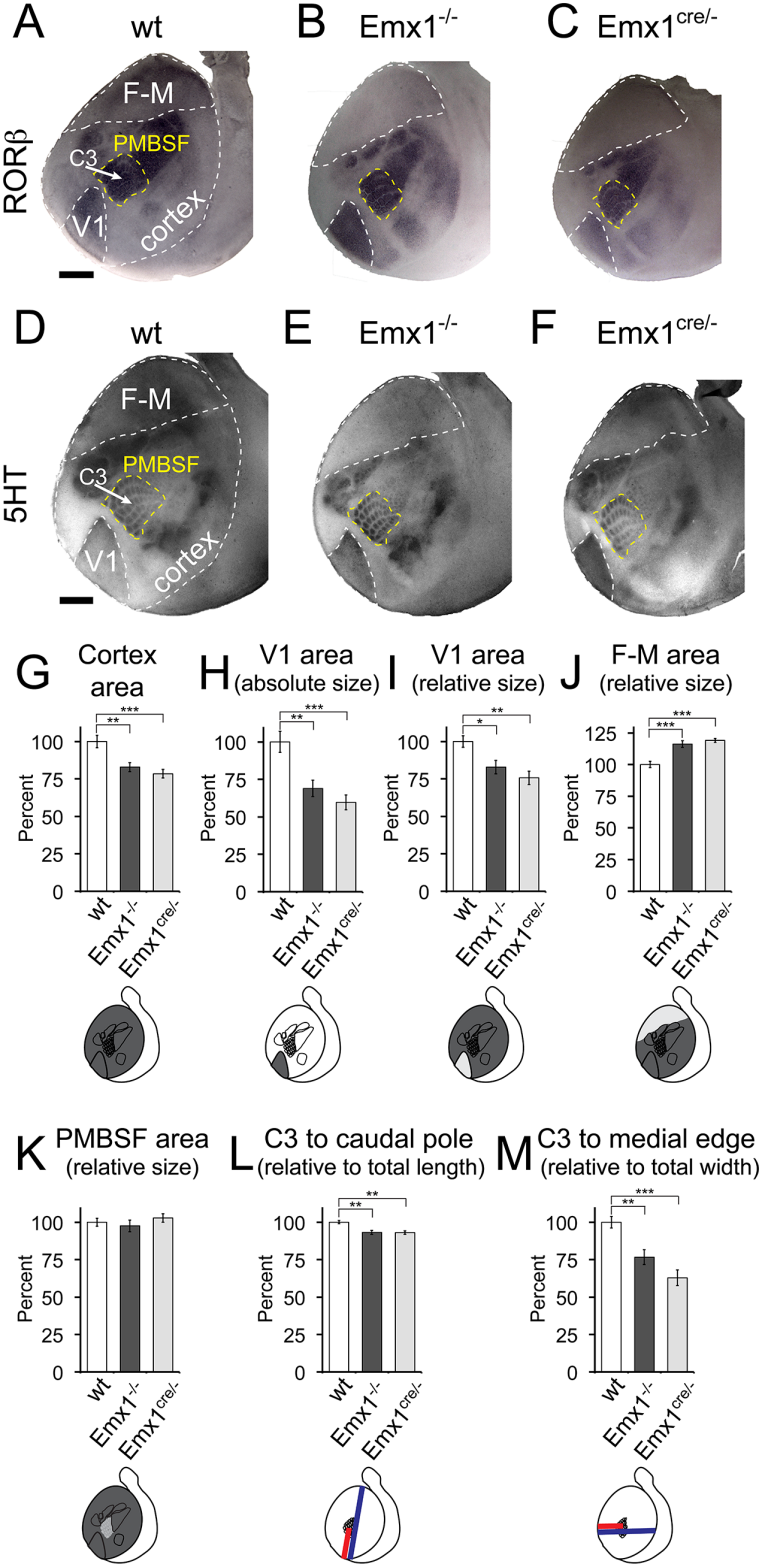
Thalamic terminations demonstrate changes in primary area size in tangential sections following Emx1 deletion. RORβ is expressed in layer 4 of sensory areas shown here on tangential sections of flattened P7 cortices of wt (A), Emx1^-/-^ (B), and Emx1^cre/-^ (C) animals. The changes observed in tangential sections were the same seen in the sagittal plane (Fig 1) and WMISH (Fig 2). (D–F) 5HT immunostaining highlighting thalamic terminations on tangential sections of flattened P7 cortex. 5HT staining was utilized to examine area patterning changes in thalamocortical innervation, which were the same as those seen by RORβ expression (A–C). All quantifications were performed using 5HT stained sections. (G) Total cortical area was measured (outlined in wt sections, gray in schematic), and both Emx1^-/-^ and Emx1^cre/-^ 5HT stained cortices were significantly smaller than wt. (H) V1 area (outlined in sections, gray in schematic) was significantly smaller in both Emx1^-/-^ and Emx1^cre/-^ cortices than wt. (I) V1 area (outlined in sections, light gray in schematic) was normalized by total cortical area (dark gray in schematic), and V1 area relative size in both Emx1^-/-^ and Emx1^cre/-^ cortices was significantly smaller than wt. (J) F-M area (outlined in sections, light gray in schematic) was normalized by total cortical area (dark gray in schematic), and F-M area relative size in both Emx1^-/-^ and Emx1^cre/-^ cortices was significantly larger than wt. (K) PMBSF (outlined in sections, light gray in schematic) was normalized by total cortical area (dark gray in schematic), and PMBSF area relative size was the same between all genotypes. (L) The length from the C3 barrel to the caudal pole (red line in schematic) was normalized by total cortical length (blue line in schematic), and relative C3 length in both Emx1^-/-^ and Emx1^cre/-^ cortices was significantly smaller than wt. (M) The width from the C3 barrel to the medial edge (red line in schematic) was normalized by total cortical width (blue line in schematic), and the relative C3 width in both Emx1^-/-^ and Emx1^cre/-^ cortices was significantly smaller than wt. Data presented as percentage of wt measurements. V1, primary visual area; PMBSF, posteromedial barrel subfield of the primary somatosensory area; arrow points to C3 barrel location within PMBSF; F-M, frontal and motor areas. Medial to the left, anterior to the top. *P<0.05; **P<0.01; ***P<0.001; Tukey’s HSD post-hoc test. Scale bar, 1.0 mm. Bar in A applies to A–C; bar in D applies to D–F.

RORβ expression viewed in tangential sections recapitulates the results reported above by WMISH and in sagittal sections. RORβ expression revealed in Emx1 mutants that V1 was reduced in size, F-M was expanded, while S1 was shifted posterio-medially (Fig 3A–3C). These gene expression results were mirrored by thalamocortical terminations revealed with 5HT immunostaining. 5HT staining also demonstrated qualitatively that V1 was reduced in size, F-M was expanded, while S1 was shifted posterio-medially (Fig 3D–3F).

Total cortical size was quantified in these 5HT tangential sections, and just as in WMISH approaches, we found that the differences in cortical size between the genotypes were statistically significant (F_(2,22)_ = 10.88; P = 0.0005; wt n = 8; Emx1^-/-^ n = 9; Emx1^cre/-^ n = 8) (Fig 3G). Specifically, Emx1^-/-^ cortices were 17.08 ±3.07% smaller than controls (P = 0.0044), and Emx1^cre/-^ cortices were 21.56 ±2.91% smaller than controls (P = 0.0006) (Fig 3G). However, there were no significant differences between the Emx1 deletions (P = 0.6205) (Fig 3G).

There was an absolute reduction in V1 size measured by 5HT expression in tangential sections that was significantly different between the genotypes (F_(2,22)_ = 12.50; P = 0.0002) (Fig 3H). 5HT expression demonstrated that V1 size was reduced 31.07 ±5.42% in Emx1^-/-^ (P = 0.0029) and 40.36 ±5.07% in Emx1^cre/-^ (P = 0.0003) compared to controls, while there were no differences between the Emx1 deletions (P = 0.5084) (Fig 3H). As in WMISH quantifications, area size was also normalized by the total cortical size in 5HT measurements to correct for any differences due to the observed changes in total cortical size yielding the relative size of an area. The relative V1 size measured by 5HT expression in tangential sections was significantly different amongst genotypes (F_(2,22)_ = 8.10; P = 0.0023) (Fig 3I). 5HT expression demonstrated that the relative V1 size was reduced 17.12 ±4.44% in Emx1^-/-^ (P = 0.0249) and 24.24 ±4.50% in Emx1^cre/-^ (P = 0.0021) compared to controls, while there were no differences between the Emx1 deletions (P = 0.4764) (Fig 3I). The relative F-M size as measured by 5HT expression was also significantly different amongst genotypes (F_(2,22)_ = 18.84; P < 0.0001) (Fig 3J). 5HT expression demonstrated that relative F-M size was increased 16.16 ±2.71% in Emx1^-/-^ (P = 0.0002) and 19.10 ±1.63% in Emx1^cre/-^ (P < 0.0001) compared to controls, while there were no differences between the Emx1 deletions (P = 0.6477) (Fig 3J). Conversely, the relative size of the posteromedial barrel subfield portion of S1 (PMBSF) was 2.41% ±3.96% smaller in Emx1^-/-^ and 2.86% ±2.88% larger in Emx1^cre/-^ than controls (F_(2,22)_ = 0.649; P = 0.532), neither of which approached statistical significance (Fig 3K).

To quantify the observed shift of primary sensory areas, the distance from the caudal pole of the neocortex to the C3 barrel in the PMBSF was compared to the total length of the neocortex. This relative C3 length measured in 5HT-stained tangential sections was significantly different amongst genotypes (F_(2,21)_ = 11.33; P = 0.0005) (Fig 3L). 5HT expression demonstrated that the relative C3 length was reduced 6.83 ±1.33% in Emx1^-/-^ (P = 0.0014) and 6.93 ±1.17% in Emx1^cre/-^ (P = 0.0013) compared to controls, while there were no differences between the Emx1 deletions (P = 0.9981) (Fig 3L). The distance from the medial edge of the neocortex to the C3 barrel in the PMBSF was compared to the total width of the neocortex. This relative C3 width measured in 5HT-stained tangential sections was significantly different amongst genotypes (F_(2,22)_ = 15.65; P < 0.0001) (Fig 3M). 5HT expression demonstrated that the relative C3 width was reduced 23.33 ±5.00% in Emx1^-/-^ (P = 0.0032) and 37.13 ±5.28% in Emx1^cre/-^ (P < 0.0001) compared to controls, while there were no significant differences between the Emx1 deletions (P = 0.1561) (Fig 3M).

The measurements from 5HT immunostained flattened tangential sections confirmed the results observed utilizing whole mount in situ hybridization demonstrating that V1 was reduced, F-M was expanded, and that areas were shifted posterio-medially in Emx1 deletions. The magnitude of the quantitative results were consistent between whole mount and flattened approaches and between the two genetic deletion lines.

Projections from primary thalamic sensory nuclei project to the same sensory modality within the neocortex, and do not send projections to other sensory modalities [2,42]. Visual information from the periphery is relayed to the neocortex by thalamocortical afferents projecting from the visual thalamic nucleus, the dorsal lateral geniculate (dLG) [43]. Thus since the dLG receives visual input, it will project to V1 in the occipital cortex and not to any other primary sensory areas.

**Fig 4:**
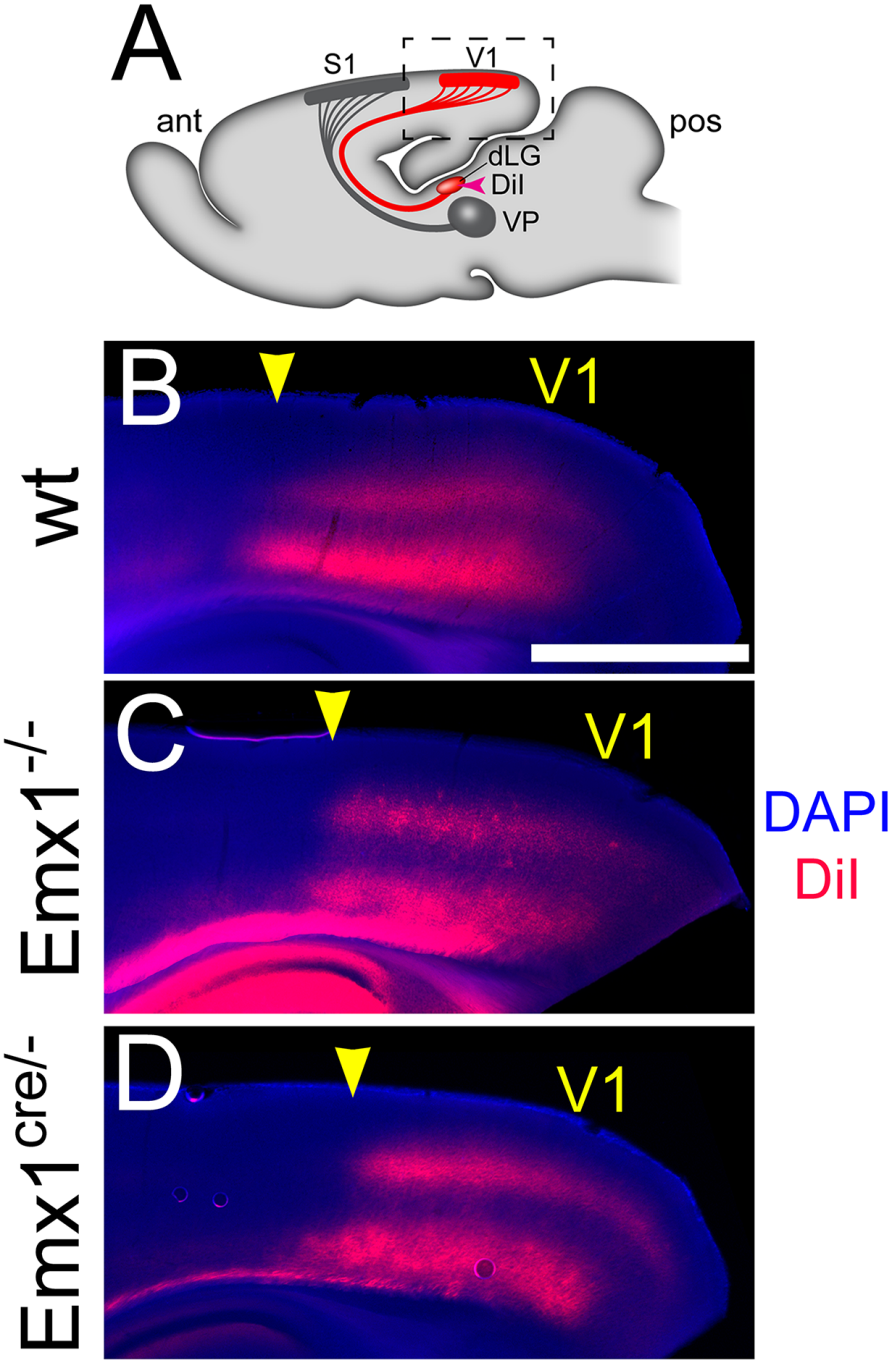
Geniculocortical projections to V1 correctly match the reduced size of V1 in Emx1 deletions. Labeling of geniculocortical projections was performed by placing DiI crystals into the primary visual thalamic nucleus (dorsal lateral geniculate, dLG). (A) Sagittal schematic of DiI label placement (pink arrowhead) and visual sensory connections between cortex and thalamus (red), as well as somatosensory connections between cortex (S1) and thalamus (ventral posterior nucleus, VP) in gray. The highlighted region in this schematic is presented in subsequent panels. Sagittal sections through wt (B), Emx1^-/-^ (C), and Emx1^cre/-^ (D) brains, where the distribution of DiI labeled geniculocortical projections is shown in red. Tissue has been counterstained with DAPI (blue) to reveal gross anatomic structure of the sections. The distribution of labeled projections in both Emx1 deletions reveal that V1 was reduced and its anterior border (arrowhead) has been shifted posteriorly. Labeling also showed that geniculocortical projections from the dLG correctly targeted the reduced V1 and did not ectopically project to S1 or other sensory areas in both Emx1 deletions. V1, primary visual area; S1, primary somatosensory area; dLG, dorsal lateral geniculate (visual thalamic nucleus); VP, ventral posterior (somatosensory thalamic nucleus). Anterior is to the left, dorsal to the top. Scale bar, 1.0 mm, applies to B–D.

To investigate the connectivity between thalamus and cortex, we placed a DiI crystal into the dLG of post-mortem mature mouse brains (wt n = 10; Emx1^-/-^ n = 8; Emx1^cre/-^ n = 7). Geniculocortical afferents from the dLG projected to the occipital cortex in all of the animals examined (Fig 4). Importantly, the geniculocortical input matched the reduced V1 size in both Emx1 deletion approaches (Fig 4). Despite the posterior shift of sensory areas detailed above, geniculocortical innervation remained specific to V1 in Emx1 deleted animals. The geniculocortical input in Emx1 deletions did not invade the region of occipital cortex immediately anterior to V1, which would still be V1 in control animals. The geniculocortical afferents correctly targeted the posteriorly shifted V1 delineated by molecular markers and did not ectopically project to other sensory areas.

## Discussion

This study examined neocortical area patterning changes following Emx1 deletion. While previous studies have attempted to determine if Emx1 participates in area patterning [16,22,24], all of these studies were limited in scope because they were undertaken at perinatal time points. This is particularly problematic for area patterning studies since areas do not possess most of their defining characteristics until post-natal time points [2,3]. Unique gene expression and thalamocortical input are two critical features that can be exploited to examine and verify primary areas experimentally, both of which emerge near the end of the first post natal week [41,42]. Unlike previous studies, the present work was performed in cortices with areas that appear mature (at post natal day 7), which allowed for thorough examination of area specific gene expression as well as differentiated and refined thalamocortical input.

These findings in Emx1 deleted animals demonstrated that V1 size was reduced as measured by the expression of established marker genes. These marker genes also showed that F-M was expanded and that S1 was shifted posterio-medially in Emx1 deleted animals. The magnitude of these changes was consistent between both whole mount preparations and flattened sections, as well as between Emx1 deletions. Thalamic input demonstrated the same changes in area patterning shown by gene expression in Emx1 deletions. Importantly, the laminar expression of the marker genes was normal and appropriate in the Emx1 deletions. Thalamic projections were also to the appropriate primary sensory cortical targets in Emx1 deleted animals with no ectopic projections detected.

**Fig 5:**
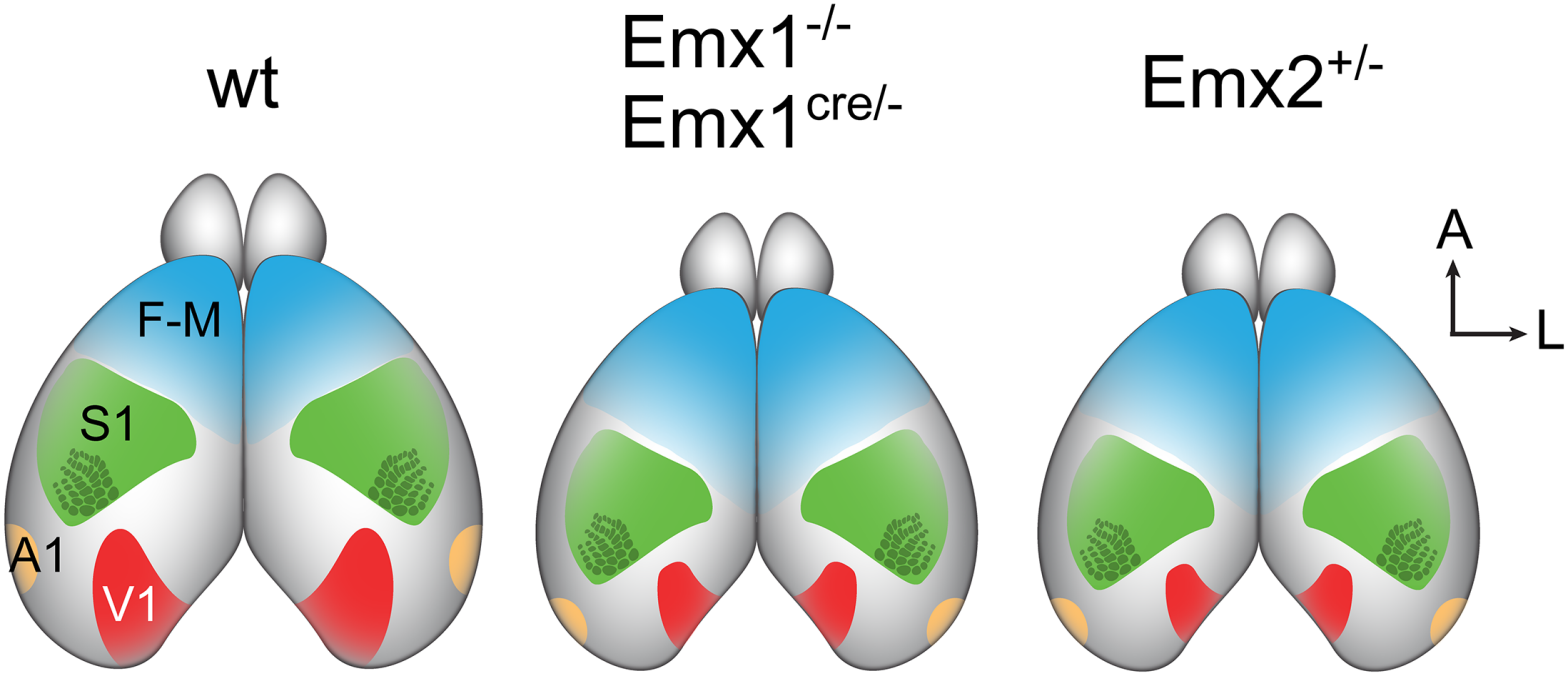
Summary of neocortical area patterning changes following Emx1 deletion. Schema of primary sensory and motor areas in the wild type neocortex (control), the homozygous Emx1 deletion neocortex (both approaches), and the heterozygous Emx2 deletion neocortex. All of the data presented in this study have demonstrated that following Emx1 deletion, V1 was reduced in size and F-M was expanded, while S1 was shifted posteriorly. This patterning phenotype was observed in both the Emx1^-/-^ and Emx1^cre/-^ mouse lines. These changes were very similar to those observed in Emx2 heterozygous mutants [13]. Our results demonstrate that Emx1 is an intrinsic area patterning transcription factor and is required for V1 specification. A, anterior; L, lateral; V1, primary visual area; S1, primary somatosensory area; A1, primary auditory; F-M, frontal and motor areas.

Neocortical areas shifted posterio-medially in Emx1 deletions consistent with the highest level of Emx1 expression, which is expressed in a high posteromedial to low anterolateral gradient [18,19]. The area located near the highest level of Emx1 expression, V1, was also the area that is most effected by the deletion and the area reduced in size. In previous studies where an intrinsic area patterning TF has been manipulated in the developing neocortex, thalamocortical innervation matched the shifts in area location and size observed using molecular markers [13,15]. Emx1 deleted animals possessed thalamocortical projections that were appropriately targeted despite shifts in area location, and also lacked ectopic projections. The area patterning phenotype yielded by the Emx1 deletions was consistent with the phenotypes observed following cortex specific deletion of other area patterning TFs [15,17,44]. Furthermore, the presented Emx1 changes were consistent across the markers utilized, across the preparations utilized, and across deletion approaches. Taken together these findings demonstrated that Emx1 was required for neocortical area patterning, and was required for V1 specification (Fig 5).

Because neocortical area patterning genes are also tied to proliferation of neural precursors, it is difficult to separate a gene’s role in proliferation from fate specification. Due to the decrease in overall cortical size, proliferation clearly contributes to the observed Emx1 phenotype. However if Emx1 does not play a role in specifying sensory areas (i.e. area patterning), then the S1 and F-M areas would likely exhibit changes in size different from what we have reported. A purely proliferative effect could yield either similar increases in relative S1 and F-M size, or graded increases in relative S1 and F-M size where S1 would be slightly enlarged and F-M would exhibit a more pronounced increase. In either scenario, the combined increases should be equal to the reduction in relative V1 size to account for the decrease in occupied cortical space. This was not what was observed in the presented data, rather the relative size of S1 was unchanged (as approximated by measurements of PMBSF) (Fig 3). Instead, the increase in relative size of F-M areas solely accounted for the cortical space previously occupied by V1 (Figs 2 and 3). These changes are consistent with the area patterning changes observed in the constitutive heterozygous deletion and the cortex specific deletion of Emx2 [13,44], and thus strongly suggest that the phenotype observed in Emx1 deletions was the result of changes in area patterning.

Emx1 expression is also observed in the hippocampus [45,46]. Previous research has demonstrated that the horns of cornu ammonis (CA) are unaffected in Emx1 mutants, with a mild decrease in the size of the dentate gyrus perinatally [22], which becomes a bit more pronounced at adult stages [47]. Consistent with these prior findings we observed, via Cad8 staining, that the CA appeared normal size in Emx1 deletions. The position of the hippocampus was able to provide a relatively consistent landmark between genotypes in spite of a slight posterior shift observed in Emx1 deletions. Using this landmark in Cad8 stained sagittal sections we observed that, relative to the position of the hippocampus, V1 was smaller and shifted posteriorly in Emx1 deletions (Fig 1). While this was the only marker utilized possessing an internal landmark, given the consistency between all of the data presented (including Cad8 in both sagittal and whole mount preparations) it is not unreasonable to assume this is representative of the Emx1 deletion genotypes.

Emx1 is the first area patterning TF that belongs to the same gene family as another established area patterning TF: Emx2 [8,9,13]. Analyses of gene duplication events in fish and early chordate animals suggest that Emx2 is the older of the two genes [48]. According to the duplication-degeneration-complementation model of gene duplication preservation, the expression and function of these two genes should be complementary [48,49]. The expression patterns of both Emx genes in the neocortex are very similar, as both are in gradients with the highest level of expression located posterio-medially [19–21]. The current study, in combination with previous studies [8,9,13,14], demonstrated that the functions of both Emx genes in neocortical patterning were also quite similar. Emx2 is also required for V1 specification, and loss of Emx2 also causes a reduction in V1, an expansion of F-M, as well as a posterior shift of S1 [8,13,14,16]. Such overlap in expression pattern and function between Emx1 and Emx2 strongly suggests that their roles in area patterning are complementary.

It is also possible given that Emx2 is the older gene, expressed from an earlier time point (E8.5 vs. E9.5) [20], with a more pronounced phenotype, that Emx2 lies upstream of Emx1. Recent findings demonstrate that patterning genes expressed in post-mitotic neurons also play a role in area patterning [50,51]. Given that Emx2 is expressed only in progenitors while Emx1 is expressed in both progenitors and neurons [46], the non-redundant role Emx1 plays in area patterning may be in post-mitotic neurons. The presented work detailed constitutive deletions of Emx1, therefore our data is unable to provide any insight into the potential timing of Emx1’s effect on area patterning. It should also be noted that previous studies have not indicated that either Emx gene regulates the expression of the other [4], but this of course does not rule out the possibility of indirect regulation.

While several studies have explored the potential targets of Emx2 [52–54], there is a dearth of investigation into the targets of Emx1, and Emx1 function in general. To date only one regulatory target of Emx1 has been characterized, Neuropilin 1 (Nrp1) [55]. Nrp1 is the receptor of the axon guidance cue semaphorin 3A, and these guidance cues have been previously demonstrated to participate in thalamocortical pathfinding [56,57]. Thalamocortical pathfinding also plays an important role specifying primary sensory areas [6]. However, how area patterning transcription factors potentially modulate thalamocortical input is not understood and much remains to be uncovered. Furthermore, it is also unknown whether Nrp1 has any Emx1 dependent effect on thalamocortical innervation and whether this potential regulatory effect holds any influence on area patterning.

There may also be some functional redundancy between Emx1 and Emx2, however the possibility that these two Emx genes are the only genes required for the specification of posteriomedial areas, in particular V1, is rather intriguing. While previous studies have examined potential cooperative roles for the Emx genes in area patterning [16,24], these too suffered from the same shortcoming as previous Emx1 studies: they were performed at perinatal time points prior to the emergence of mature area features. It is now clear that both Emx genes are area patterning genes, revisiting this question in animals with mature area features is the next logical step. These future studies will further enhance our understanding of the intrinsic mechanisms that specify functional neural areas.

## Supporting Information

S1 Appendix Dataset of measurements collected from whole mount and flattened stains. (XLS)
